# Gene modification strategies using AO‐mediated exon skipping and CRISPR/Cas9 as potential therapies for Duchenne muscular dystrophy patients

**DOI:** 10.1049/enb.2020.0017

**Published:** 2020-12-09

**Authors:** Marthe Helene Solberg, Maryam Shariatzadeh, Samantha L Wilson

**Affiliations:** ^1^ National Centre for Sport and Exercise Medicine, School of Sport Exercise and Health Sciences Loughborough University Epinal Way, Loughborough Leicestershire LE11 3TU UK; ^2^ Centre for Biological Engineering, Wolfson School of Mechanical, Electrical and Manufacturing Engineering Loughborough University Epinal Way, Loughborough Leicestershire LE11 3TU UK

**Keywords:** patient diagnosis, cellular biophysics, diseases, muscle, cardiology, microorganisms, molecular biophysics, genetics, neurophysiology, biochemistry, gene therapy, genomics, gene modification strategies, AO‐mediated exon skipping, Duchenne muscular dystrophy patients, genetic disease, patients experience muscle weakness, reduced life expectancy, developed genetic editing strategies aim, severe DMD phenotypes, milder disease course, antisense oligonucleotide‐mediated exon, dystrophin protein expression, potential therapeutic benefits, gene‐editing strategies, therapeutic benefit, long‐term efficacy, exon skipping drugs, adeno‐associated viral‐delivered CRISPR‐Cas9 gene editing, adeno‐associated viral‐delivered clustered regularly interspaced short palindromic repeat

## Abstract

Duchenne muscular dystrophy (DMD) is an X‐linked genetic disease affecting 1 in 5000 young males worldwide annually. Patients experience muscle weakness and loss of ambulation at an early age, with ∼75% reduced life expectancy. Recently developed genetic editing strategies aim to convert severe DMD phenotypes to a milder disease course. Among these, the antisense oligonucleotide (AO)‐mediated exon skipping and the adeno‐associated viral‐delivered clustered regularly interspaced short palindromic repeat (CRISPR) associated protein 9 (adeno‐associated viral (AAV)‐delivered CRISPR/Cas9) gene editing have shown promising results in restoring dystrophin protein expression and functionality in skeletal and heart muscle in both animals and human cells in vivo and in vitro. However, therapeutic benefits currently remain unclear. The aim of this review is to compare the potential therapeutic benefits, efficacy, safety, and clinical progress of AO‐mediated exon skipping and CRISPR/Cas9 gene‐editing strategies. Both techniques have demonstrated therapeutic benefit and long‐term efficacy in clinical trials. AAV‐delivery of CRISPR/Cas9 may potentially correct disease‐causing mutations following a single treatment compared to the required continuous AO/PMO‐delivery of exon skipping drugs. The latter has the potential to increase the dystrophin expression in skeletal/heart muscle with sustained effects. However, therapeutic challenges including the need for optimised delivery must be overcome in to advance current clinical data.

## 1 Introduction

Duchenne muscular dystrophy (DMD) is a fatal X‐linked recessive disease that affects 1 in 5000 male births worldwide [1, 2, 3], and 2500 people are currently living with DMD in the UK according to the UK National Health Services (NHS) [4]. DMD is the most severe form of muscular dystrophy, and life expectancy is ∼25 years [5]. Disease onset emerges in patients from 2–5 years of age and manifests with early walking difficulties. This results in a gradual loss of ambulation until most patients become wheelchair‐bound by the age of 12 years [6]. Degeneration of myofibres leads to skeletal muscle deformities, adipose tissue replacement, respiratory issues, and cardiomyopathy, and ultimately, death [2]. In contrast, Becker muscular dystrophy (BMD) is a milder phenotype of the muscular dystrophies, caused by genome deletions that do not disrupt the reading frame and presents milder symptoms compared to DMD and thus increases patient life expectancy [5].

DMD biopathology is caused by single or multiple mutations or deletions of the exons along the dystrophin gene, that causes disruptions in the reading frame of the precursor messenger RNA (pre‐mRNA), resulting in the production of an absent or shortened dystrophin protein [7]. Dystrophin is the longest known gene within human DNA, consisting of 79 exons and 2.6 million base pairs, thus contributing to the high rate of spontaneous mutations, deletions and duplications observed [8, 9]. Approximately 4000 different mutations and deletions have been reported in the dystrophin gene, of which, ∼60% are located within the proximal exon region 1–22 and in the distal region 45–55 [8]. Dystrophin is a scaffolding protein that attaches the muscle filament cytoskeleton (actin) to the extracellular matrix and cell membrane as a supporting structure that maintains the integrity of the muscle cell through the dystrophin‐associated glycoprotein complex [7]. The presence of a shorter or non‐existent dystrophin protein causes fragility to the cell membrane, which impairs cell regeneration and initiates myocyte necrosis. These events eventually lead to the replacement of the contractile filaments with fatty and fibrotic tissue [3, 7]. This results in rupture of the cell membrane as the myofilaments contract, releasing creatine kinase (CK) and calcium ions (Ca + ) into the extracellular matrix and subsequently into the bloodstream. Therefore, blood CK levels are currently used to diagnose muscular dystrophies from a young age [10].

**Fig. 1:**
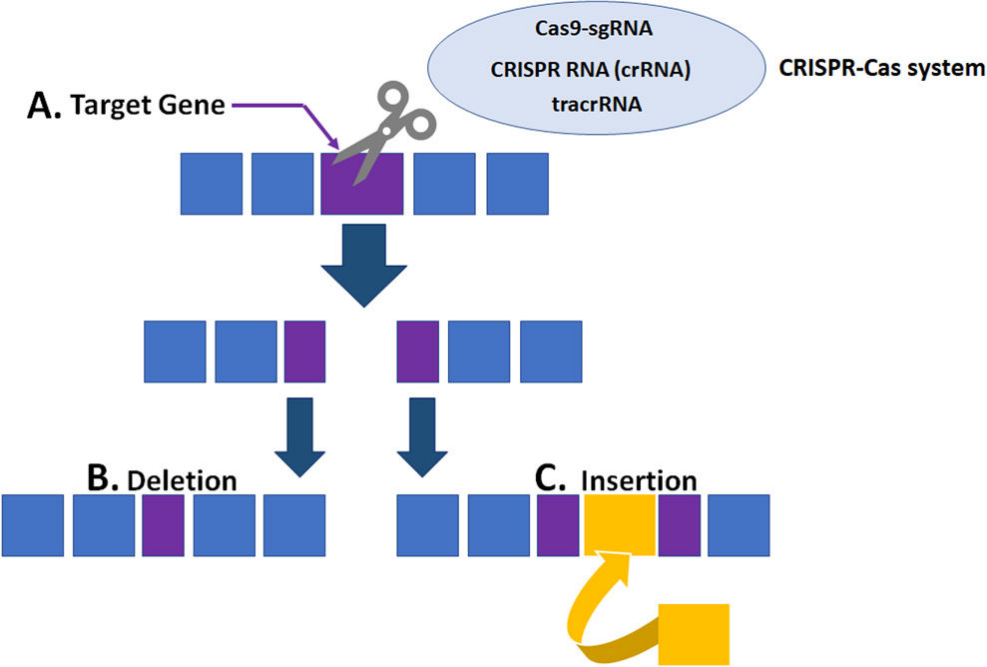
Genome editing/modification by CRISPR‐Cas9 ***(a)*** Principle of gene editing is the cleavage of the double‐stranded DNA at a targeted position on the genome, ***(b)*** Trans‐activating CRISPR RNA (tracrRNA) brings the Cas9 nuclease to the target site, which binds with the CRISPR RNA (crRNA) with the homologous sequence. Once the Cas9‐sgRNA complex cleaves the target gene the Cas system modifies the function of the target gene via a deletion mutation, ***(c)*** Cas system modifies the function of the target gene via insertion mutation. The CRISPR‐Cas repair process offers an efficient practical genomic editing technique that can be used to treat a variety of genetic diseases including DMD

Recently, gene‐editing therapies have been developed as a potential treatment of DMD by restoring the reading frame of the dystrophin protein [11]. The transcription of dystrophin may be recuperated via the skipping or deletion of one or more sections in the pre‐mRNA or DNA. This results in the production of a truncated but potentially functional dystrophin protein referred to as micro‐dystrophin, which requires a minimum of 1001 amino acids to achieve functionality [12]. It is estimated that 60–80% of DMD patients could benefit from gene‐editing therapies such as pre‐mRNA exon skipping or clustered regularly interspaced short palindromic repeats (CRISPR) DNA modification or deletion [3]. These approaches include adeno‐associated viral delivery (AAV) for DNA‐targeted editing or deletion (Fig. 1), and antisense oligonucleotide (AO)‐mediated exon skipping of mRNA structures.

The aim of this narrative review and critique the current literature regarding the efficacy, safety and clinical progress of two prevailing gene modification strategies: (i) AO‐mediated exon skipping and (ii) genome editing/deletion by CRISPR/Cas9 complex and discuss the potential therapeutic benefit to patients suffering from DMD.

## 2 AO‐mediated exon skipping

**Fig. 2:**
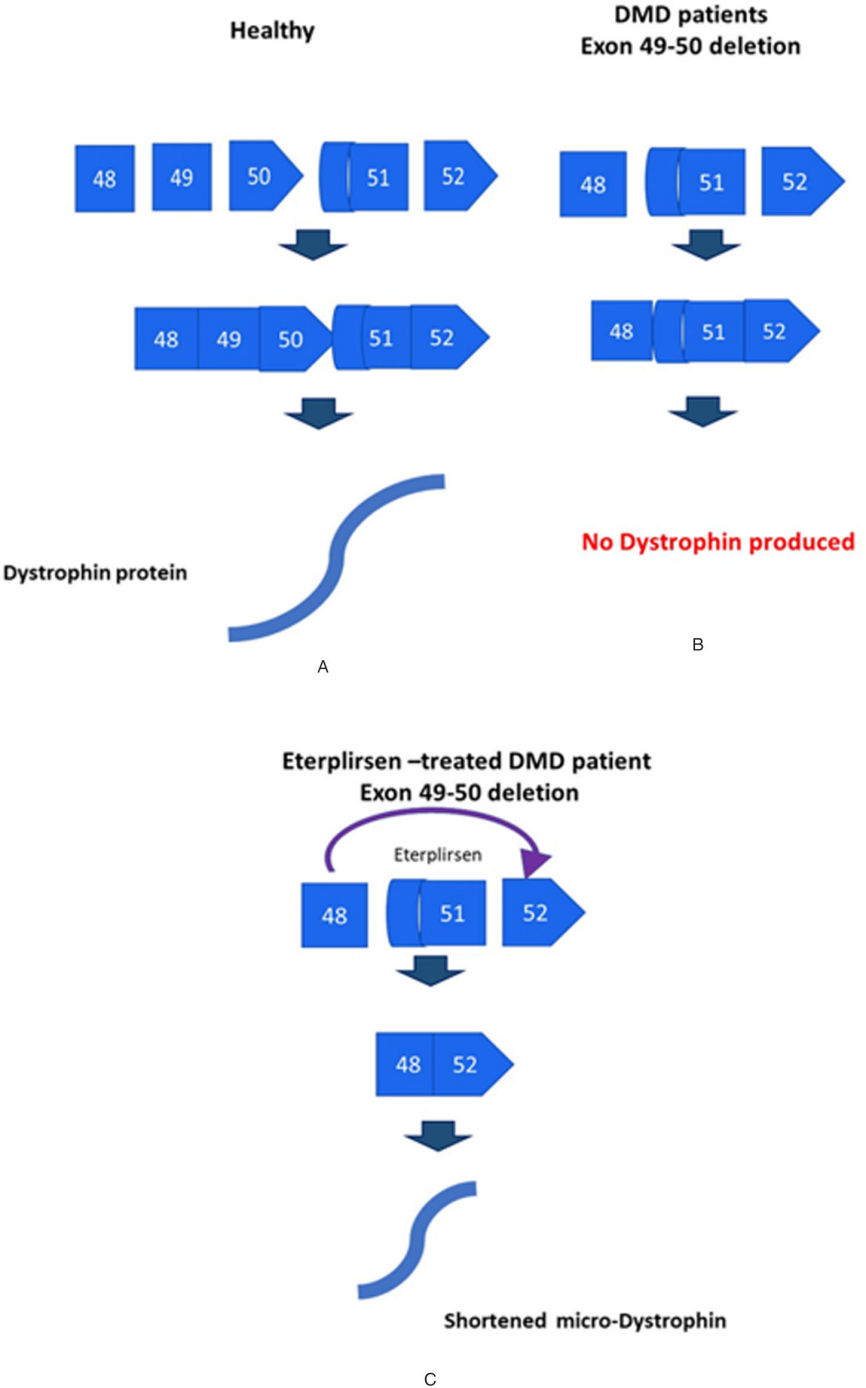
Singular exon‐skipping mechanism of exon 51 by ***(a)*** Eteplirsen drug treatment creates shorter, potentially functional dystrophin protein, ***(b)*** DMD deletion of exon 49–50, ***(c)*** DMD deletion of exon 49–50 causes a frameshift in the exon sequence, resulting from a premature stop codon between exon 48 and 51. Adapted from: Lim *et al*. [17]

The conventional approach to gene‐editing therapies involves skipping a single exon in the dystrophin pre‐mRNA, thus creating alternate splicing that bypasses the mutated exons by eliminating the premature stop codon produced by the out‐of‐frame mutations [6, 13]. Exon skipping is achieved when a single‐stranded neutrally charged AO drug, similar in structure to RNA, binds to the pre‐mRNA and simply skips the exons during translation of the mature messenger‐RNA (m‐RNA) by the ribosome [8, 14]. In 2016, Eteplirsen (branded EXONDYS 51®, by Sarepta Therapeutics [15]) was the first AO‐drug to receive accelerated approval from the American Food and Drug Association (FDA) to be used in human trials of patients with DMD amenable to skipping exon 51 (exon 49 and 50 deletions), by the restoration of the reading frame between exons 48 and 52 (Fig. 2) [16].

This single exon skipping approach has estimated applicability to 14% of all patients with DMD [17], leaving the majority of patients unsusceptible to treatment and without other treatment options. Concerns have been raised regarding Eteplirsen due to its clinical efficacy of <2% rescued dystrophin following 180 weeks of treatment compared to historical controls, and lack satisfactory improvement in patient ambulation [17]. Thus, results from an ongoing stage 3 clinical trial from Sarepta Therapeutics [18], due 2020, will be the concluding factor to receive full FDA approval. More recently, early‐stage clinical trials have successfully achieved skipping of multiple exons in ‘hotspot’ regions of murine, canine, and humanised mouse models [8, 19, 20, 21], with the aim of transforming DMD to the milder BMD phenotype. The target regions have been established from studies of patients with naturally occurring deletions of exon regions 3–9 [22] and 45–55 [23, 24], living with an asymptomatic or mild disease course. Despite promising advances in AO‐mediated exon skipping for DMD, some challenges remain for future development and to become a common strategy for treating DMD patients, such as the need for continuous weekly or biweekly administration to maintain therapeutic benefit. Due to its natural composition and rapid clearance from skeletal muscle, repeated administration would be inevitable. In addition, the long‐term efficacy of Eteplirsen is reliant upon the development of modified PMO (synthetically made AO cocktails with a phosphorylated backbone) to target specific exon regions and enhance cell permeability [19, 21, 25]

## 3 Gene modification strategies: CRISPR/Cas9

In contrast to AO‐mediated exon skipping, CRISP and Cas9 nuclease proteins merged with a guide RNA (gRNA) structure has the potential to directly correct disease‐causing genetic mutations in patients with DMD with a single treatment [26]. The bacterial CRISPR/Cas9 system consists of a nuclease protein Cas9 that induces a double‐stranded break along the DNA genome as directed by a gRNA to locate the Cas9 to the desired DNA mutation spot, inducing a cell repair response [27]. Restoration of the cut DNA‐sequence occurs through two mechanisms: (i) homology‐directed repair (HDR), with a DNA template (DNAt) delivered with CRISPR/Cas9 to replace the cut sequence with non‐mutated wild‐type DNA or by (ii) non‐homologous end‐joining (NHEJ), where a natural repair response is initiated in the cell by split end joining [27]. Delivery methods of the CRISPR/Cas9 complex depend on the proliferative status of the cell, meaning the cells ability to accept an added DNAt. Akin to exon skipping strategies, CRISPR‐Cas9 may have the potential to delete the disease‐causing genome mutations associated with severe disease phenotypes [12].

## 4 Comparison of the efficacy of exon‐skipping and CRISPR‐Cas9 gene modification strategies

As demonstrated from previous clinical trial data [28], antisense PMO provides promising chemistry for exon‐skipping therapy, especially due to the safety and efficacy in skeletal muscles. The development of specific, targeted AO sequences (cocktails) can significantly increase the therapeutic effect by skipping multiple exons along the pre‐mRNA. However, little effect has been reported with systemic intravenous delivery of PMOs on the myocardium of DMD animal models, which remains a challenge to prove the therapeutic benefit as this is the main cause of death in human patients [5]. Recently, Echigoya *et al.* (2017) utilised an arginine‐rich cell‐penetrating conjugated morpholino to successfully restore cardiac function in canine X‐linked dystrophic (CXMD) dogs by skipping exons 6 and 8, with spontaneous splicing of exon 9 [19]. Intravenous, intramuscular and coronary injections into dystrophic CXMD dogs restored dystrophin expression in the myocardium as well as in skeletal myofibres and showed electrocardiogram abnormalities (*Q* ‐amplitude and *Q* /*R* ratio) improvement after intracoronary or intravenous injections with a dosage of 12/mg/kg across four systematic injections. The Western blotting analysis confirmed dystrophin expression ∼5% compared to healthy wild‐type dogs. Similarly, Yokota *et al.* [29] detected modest dystrophin expression in cardiac muscles in dogs administered with higher dosages of PMOs: 40 mg/kg and 66 mg/kg, respectively.

However, the findings of these studies were reported as a percentage of natural history controls, and no control group was employed in the study. Similarly, low expression of dystrophin in cardiac muscle was observed in a muscular dystrophy exon 52 (mdx52) murine model after long‐term systemic PMO skipping of exons 45–55 [30]. It is worth noting that dystrophin expression of 1–2% improves cardiac function and may therefore have a therapeutic effect regardless of modest expression and is likely due to lower diffusion and permeability of the cell membranes in the heart [29]. Significant increase of genome deletion in cardiac muscles over time in systemically treated mice, concludes estimations of slower turnover of cardiomyocytes compared to skeletal myocytes [31] and may explain the modest changes in cardiac dystrophin expression observed in other studies of shorter duration. However, the difference in anatomy and physiology of animal heart muscles compared to humans may not translate these therapeutic benefits to DMD patients, and long‐term dystrophin expression across years lived has not yet been established in animals or in humans with DMD.

Permanent removal of a disease‐causing mutation in exon 23 by NHEJ has been reported in mdx mice using an in vivo myo‐editing model [32]. The continued increase in dystrophin positive fibres in mouse cardiomyocytes occurred between 3 and 12 weeks post‐AAV‐delivery of CRISPR‐Cas9, ranging from 3.2 to 9.6% 12 weeks post‐retro‐orbital and intraperitoneal injections, suggesting the efficacy of dystrophin production in the heart to increase over time. Similar results were reported with functional recovery of dystrophin in skeletal and cardiac muscles using the same technique (NHEJ) following a six‐month follow‐up study in mdx23 mice [12]. These show promising results for further development of larger mammal studies. A recent study reported the successful editing of the underlying DMD disease mutation in an engineered human heart muscle model using CRISPR/Cas9 in vitro [9]. Human cardiomyocytes are believed to be long‐lived and myocyte turnover is estimated to ∼1% per year [31], thus the potential for sustained dystrophin expression in the heart muscle seem positive.

## 5 Critique of gene modification strategies in animal models and clinical translation

### 5.1 Animal models

Mouse models for genome editing via homologous recombination of Exon 53 with dual AAV‐6 vector delivery methods have confirmed restoration of dystrophin expression and improvement of skeletal muscle function in myogenic cells [12, 32, 33, 34]. Disease progression of the mdx mouse is milder in comparison to humans and has estimated shortened lifespan of ∼25% of wild type, which is not comparable to humans, whose lifespan is shortened by ∼75%. Necrosis, fibre regeneration with centrally located nuclei and elevated levels of serum CK is evident between 4 and 6 weeks of age. At this point, the disease progression slows down, and the patient does not develop sclerosis, muscle wasting and cardiomyopathy until much later at ∼12–15 months of age [3]. Therefore, the disease course of mice is not comparable to the human disease progression, except from the extensive manifestation in the diaphragm muscle, and therapeutic effects cannot be established from murine models alone.

Despite the challenges that the industry is currently facing to develop HRD‐based therapeutics Lee *et al.* [35] reported the development of a delivery vehicle composed of gold nanoparticle DNA conjugates which enhance the on‐target efficacy of CRISPR‐Cas 9 systems. This delivery method was applied to deliver Cas9 ribonucleoprotein and donor DNA to a variety of cell types including HEK 293 embryonic stem cells, human‐induced pluripotent stem cells, and mdx mice models both in vitro and in vivo. Their results indicated that CRISPR‐Gold has the great potential to correct DNA mutation back to the wild type sequence and to restore fully functional dystrophin, without the use of viruses [35]. Tissue stem cell‐based therapies hold great therapeutic potential for the treatment of DMD and other muscle disorders [36]. In a 2017 study, Zhu *et al.* [37] described how a fibrin‐gel culture system can be used to selectively expand muscle stem cells isolated from mice skeletal muscle cells to develop a mouse model of DMD. Their results indicated that CRISP/Cas9‐based genome editing via HDR corrected the dystrophin mutation while restoring the expression of dystrophin [37].

Unlike the mdx models, affected dogs show comparable pathology progression to humans with a similar onset of muscular atrophy, fibrosis, cardiomyopathy and limb weakness [3]. This makes X‐linked canine dog models (CXDM) more suitable for preclinical trials for proving the long‐term efficacy of CRISPR/Cas9 genome editing in humans. Ensuring safety and efficacy in larger mammals are essential steps towards clinical translation of these gene‐editing approaches [38]. Recently, a novel canine model was developed, DeltaE50‐MD, replicates the severity of pathology as found in human disease expression [39], carrying a mutation that lies within the common human hotspot (Exon 50), thus providing an improved model for future human clinical trial translation. Gene editing via CRISPR and transcription activator‐like effector nucleases has been recently reported to restore dystrophin expression via HDR in myoblasts/myotubes or via intramuscular injection of golden retriever muscular dystrophy dogs. The results of this study revealed that editing methods must be further modified in order to boost the efficiency of HDR‐mediated gene repair and protein expression [40].

The majority of animal studies have reported short‐term restoration of dystrophin, typically assessed between 4 and 8 weeks [32, 41] and up to six months post‐treatment [12]. To date, one study has investigated the longitudinal effect of CRISPR/Cas9 AAV administered dystrophin restoration. Dystrophin protein was restored in the mdx‐23 mouse model for one year following single intravenous administration [38]. Due to immunogenic responses in adult mice to AAV administered CRISPR/Cas9, administration at the neonatal stage proved to be vital to the therapeutic outcome and emphasises the importance of commencing treatment in early years of living. However, considering the differences in phenotype expression in mice compared with human counterparts, it is still unclear how these findings relate to new‐born humans.

### 5.2 Translation of gene‐editing techniques

The use of AAVs in CRISPR/Cas9 delivery has presented low toxicity, immunogenicity, minimal integration risk and long‐term transgene expression, making it suitable for delivery of the CRISPR/Cas9 components in vivo [3, 31, 42]. However, human immune host response to CRISPR‐AAV needs to be carefully considered for clinical development as the pre‐existing adaptive immune response to bacteria‐derived Cas9 proteins have been reported in a recent study [43]. Regardless, AAV‐delivery of gene replacement therapy was recently approved by the FDA for treatment of inherent retinal disease [44, 45] (Spark Therapeutics [46]) and announced the first approval for AAV‐CRISPR therapy in spinal muscular atrophy (AveXis) in May 2019 [47, 48]. Currently, ongoing human trials are being conducted in spinal muscular atrophy [49], and stage 1 clinical trials in lung cancer patients [50, 51, 52, 53, 54, 55, 56] (ClinicalTrials.gov Identifier: NCT02793856), and CRISPR‐edited cancer T‐cells [57] (ClinicalTrials.gov Identifier: NCT03399448). The outcome of these studies may be the confirmation of progressing AAV‐CRISPR mediated gene editing into human clinical trials as a potential therapy for patients with DMD.

AAVs are most commonly used due to efficient permeability of the cell membrane [26]. However, the main challenges with viral delivery include its relatively small cargo capacity (<4.7 kb), and some modified Cas9 components can reach ∼4.2 kb in length [31]. Furthermore, sustained Cas9 expression in the liver and other tissues may induce complications with spontaneous mutations in undesired sites along the gene, although no significant off‐target effect has been reported in commonly predicted target sites in mice [12, 31]. By editing or bypassing sections of the dystrophin gene hotspot mutations, one CRISPR/Cas9 treatment can be applicable to ∼60–80% of patients, rather than targeting each mutated exon with specific exon‐targeted PMO cocktails [9]. This would bypass some challenges pharmaceutical companies face in providing the safety and efficacy of each individually developed AO drug and PMO cocktails to reach FDA pre‐approval. Furthermore, exon skipping can only convert DMD to the milder BMD, potentially slowing disease progression. Application of CRISPR‐Cas9 DNA targeting therapy can offer restoration of fully functional dystrophin and potentially the development of a novel curative therapy in the future.

## 6 Conclusion

DNA editing by CRIPR/Cas9 has the potential to provide patients with sustained therapeutic effects from a single delivery. Currently, HDR methods have reported the most promising evidence of restored dystrophin expression in mouse and canine models [32, 38]. However, future challenges including packaging optimised delivery methods, and safeguarding of off‐target effects have yet to be established prior to clinical acceptance [31]. Currently available AO‐induced exon skipping strategies targeting mutated sections in the pre‐mRNA remain as the fast‐tracked potentially therapeutic therapy for DMD, due to its current commercial progression. Due to low life‐expectancy in patients with DMD, the need for effective, safe, and readily available treatments continues to be the main driving factor for drug development. However, in the future years, the potential of CRISPR/Cas9 gene modification strategies may result in a favourable therapeutic outcome compared to RNA‐targeted life‐long treatment of muscular dystrophy. Genome editing represents a potentially effective treatment for eliminating the genetic cause of disease by correcting the muscle and cardiac abnormalities associated with DMD [9]. Furthermore, AAV‐CRISPR gene editing provides a potential for a single administration of CRISPR/Cas9 to have life‐long therapeutic benefits, however, this relies on the development of appropriate delivery vehicles to target desired regions without off‐target effects [58]. Although CRISPR/Cas9 genome modification processing is an exceptionally accurate, efficient, and cost‐effective, CRISPR/Cas9 as tool causes detrimental off‐target impact at the genomic level [59]. Therefore, the choice of appropriate detection tools is important as they can reduce the off‐target efficiency of CRISPR/Cas9 repair processes. These tools include (i) biased and unbiased off‐target detection with predictive on‐target and off‐target sites [60]; (ii) anti‐CRISPR proteins (CRISPR/Cas system inhibitors) [61]; (iii) CRISPR systems comprising of improved viral and non‐viral CRISPR delivery methods [62, 63].

Recently, the development of a prime editing tool including the modification of the genome's specific nucleotides without the introduction of DSBs has offered minimal off‐target efficiency in human cells, thus optimising on‐target efficacy [64]. Nevertheless, the off‐target effect limits the prime editing applications for the treatment of genetic diseases. Therefore, it is crucial that more extensive studies are completed in order to develop a ‘super CRISPR system’ that is capable of precise interpretation of off‐target efficiency. Such a system can assist in the treatment of genetic diseases whilst also being applicable to numerous clinical therapies in the biomedical and healthcare fields.

CRISPR/Cas9 has the potential advantage for DMD therapy but remains in early stages of development, and unlike AO‐mediated exon skipping, is still in the experimental stage where the further establishment of appropriate delivery, dosage, long‐term efficacy in animal and efficacy in humans are the main questions to be answered before progression into clinical translation.
